# Silicon and Water Deficit Assessment on the Cultivation of Mini Watermelon cv. Sugar Baby in Greenhouse Cultivation

**DOI:** 10.3390/plants15203107

**Published:** 2026-10-11

**Authors:** Marcio Mesquita, Viviane Correia dos Santos, Anderson Luiz Brandão, Camilla de Souza Correia, Jeffer Andrey Quintero Garcia, Maria Lucia Jaimel Verjel, Rilner Alves Flores, Moemy Gomes de Moraes, Lucas Nicioli Bandeira, Alisson Neves Harmyans Moreira, Henrique Fonseca Elias de Oliveira, Rafael Battisti

**Affiliations:** 1 Programa de Pós-Graduação em Agronomia, Escola de Agronomia, Universidade Federal de Goiás, Goiânia 74690-900, Brazil; vivianecorreia@egresso.ufg.br (V.C.d.S.); quinterojeffer@discente.ufg.br (J.A.Q.G.); luciajaime@discente.ufg.br (M.L.J.V.); rilner@ufg.br (R.A.F.); moemy@ufg.br (M.G.d.M.); alisson.harmyans@ufg.br (A.N.H.M.); 2 Programa de Pos-Graduacão Stricto Sensu em Engenharia Agrícola (PPGEA), Universidade Estadual de Goiás, Anápolis 75132-903, Brazil; brandao@aluno.ueg.br (A.L.B.); camilla.correia@aluno.ueg.br (C.d.S.C.); 3 Departamento de Botânica, Instituto de Ciências Biológicas, Universidade Federal de Goiás, Goiânia 74690-900, Brazil; lucas_bandeira@ufg.br; 4 Programa de Pós-Graduação em Irrigação no Cerrado, Instituto Federal Goiano, Ceres 76300-000, Brazil; henrique.fonseca@ifgoiano.edu.br

**Keywords:** irrigation management, efficient use of water, plant nutrition, fruit quality

## Abstract

Water deficit induces a range of morphological and physiological changes in plants, triggering multiple adaptive responses. The aim of this study was to evaluate the morphological, physiological, and postharvest responses of mini watermelon plants subjected to two foliar silicon (Si) doses and three soil water tensions during two cycles. The experimental design was in randomized blocks, in a greenhouse, with a 2 × 3 × 2 factorial scheme, with four replications. Soil water tension affected plant growth, and silicon reduced plant length by 17.5%. Fruit traits were affected by the soil water tension, with silicon influencing fruit length, weight, and rind thickness. Transpiration declined from 4.06 to 1.21 mmol m^−2^ s^−1^ in cycle 1 and from 2.79 to 1.28 mmol m^−2^ s^−1^ in cycle 2 as soil water potential decreased from −30 to −65 kPa. Biochemical parameters respond to soil water tension, with total soluble carbohydrates increasing by 75% in cycle 1 and 378% in cycle 2 as soil water tension decreased from −30 to −65 kPa in the absence of silicon application. Silicon application promoted higher total soluble carbohydrate accumulation under severe water deficit conditions. In conclusion, soil water tension was the main determinant of crop performance, while silicon showed limited effects as a drought-mitigation strategy.

## 1. Introduction

Plants are frequently exposed to environmental constraints that impose both biotic and abiotic stresses. Among abiotic factors, water deficit is particularly critical, as it induces a wide range of morphological, physiological, and biochemical responses [1]. Water deficit arises when water availability falls below crop demand [2], leading to reduced vegetative growth, accelerated leaf senescence, and decreased fruit growth rates [3]. At the cellular level, drought stress is associated with the overproduction of reactive oxygen species (ROS), largely resulting from impaired photosynthetic activity and insufficient energy dissipation [4]. Watermelon cv. Sugar Baby (*Citrullus lanatus*) is a horticultural crop belonging to the Cucurbitaceae family, characterized by high water demand throughout its growth cycle. During early developmental stages, such as the presence of two to three true leaves and the onset of flowering and fruiting, daily water consumption can reach approximately 170 mL and 250 mL per plant, respectively [5]. This crop is predominantly cultivated in semi-arid regions, where irrigation plays a crucial role in sustaining production under conditions of limited water availability [6].

In this context, silicon (Si) has emerged as a promising strategy to mitigate the adverse effects of water deficit. Si application has been shown to reduce transpiration rates without compromising cellular metabolism, thereby enhancing water use efficiency [7]. Furthermore, the deposition of Si in the cell wall promotes structural reinforcement, increasing tissue rigidity. Simultaneously, Si modulates the plant’s antioxidant defenses, stimulating their activity and contributing to protection against oxidative stress [8]. The beneficial effects of Si have been widely reported over the years. Although not classified as an essential element, it is recognized as a beneficial nutrient for several crops, particularly under stress conditions that trigger morphological and physiological adjustments. In addition, Si nutrition reduced the dependence on fungicides and pesticides [9]. As proposed by [10], Si can be considered an anomalous element: despite its non-essentiality, its presence provides substantial physiological and agronomic benefits to plants.

The response of Cucurbitaceae to Si is strongly influenced by the applied dose, Si source, application method, and stress condition. In mini watermelon, ref. [11] applied sodium silicate to leaves using 2.0 mmol L^−1^ Si once a week for four weeks and reported increases in leaf biomass, rind thickness, soluble solids, and Si accumulation under salinity, although several growth and yield traits remained unaffected. In melon, ref. [12] demonstrated that the effectiveness of potassium silicate also depends on the application route; root supply at 2 mmol L^−1^ Si induced stronger biochemical defense responses than foliar application of potassium silicate at 30 mL L^−1^. In squash (*Cucurbita pepo*), foliar applications of potassium silicate at 10, 15, and 20 g L^−1^, applied three times at weekly intervals, produced dose-dependent improvements in physiological performance, antioxidant activity, growth, and yield under water deficit, with the strongest responses at the highest concentration [13]. These contrasting protocols and responses indicate that Si effectiveness in Cucurbitaceae is highly dependent on dose and application strategy, especially under soil-water-deficit conditions.

Despite the growing interest in silicon as a potential strategy to improve plant performance under water limitation, important knowledge gaps remain. The interactive effects of silicon fertilization and soil water availability on the mini watermelon cultivar ‘Sugar Baby’ have not been reported. Moreover, the effects of Si application in Cucurbitaceae remain inconsistent, with contrasting responses according to Si concentration, source, application method, crop species, and stress intensity. These inconsistencies highlight the need for additional studies under well-defined water-deficit conditions to clarify the extent to which foliar Si can modulate physiological performance and fruit production in this crop. Therefore, this study aimed to evaluate the morphological, physiological, and fruit quality responses of mini watermelon plants grown under different soil moisture conditions combined with foliar silicon application.

## 2. Results

### 2.1. Plant Growth

The isolated factors showed that cycle, soil tension, and silicon significantly affected plant length, stem diameter, and shoot dry matter, while root dry matter showed significance for cycle and soil tension (Table 1). For the interaction factors, the pattern of significance was lower across the different plant growth parameters. Silicon showed interaction with cycle for plant length and root dry matter, and a triple interaction with soil tension and cycle for shoot dry matter. Significant interactions between cycle and soil tension were observed for stem diameter and root dry matter.

Plant length was greater in cycle 1 than in cycle 2 at both Si levels (Table 2), with values of 235 ± 51 and 186 ± 51 cm in cycle 1 compared with 132 ± 48 and 114 ± 49 cm in cycle 2 for 0 and 1.5 g L^−1^ Si, respectively. When plant length was evaluated under different soil tension levels, no significant differences were observed. Nevertheless, considering the mean across cycles and silicon treatments, plant length decreased from 188 ± 52 cm at −30 kPa to 164 ± 46 and 147 ± 62 cm under −50 and −65 kPa, respectively. Further, root dry matter and stem diameter were not affected by silicon application (Table 1). Root dry matter was greater in cycle 1, reaching 3.07 ± 1.31 g plant^−1^, whereas only 0.97 ± 0.97 g plant^−1^ was observed in cycle 2 under treatment without silicon application (Table 2).

**Figure 1:**
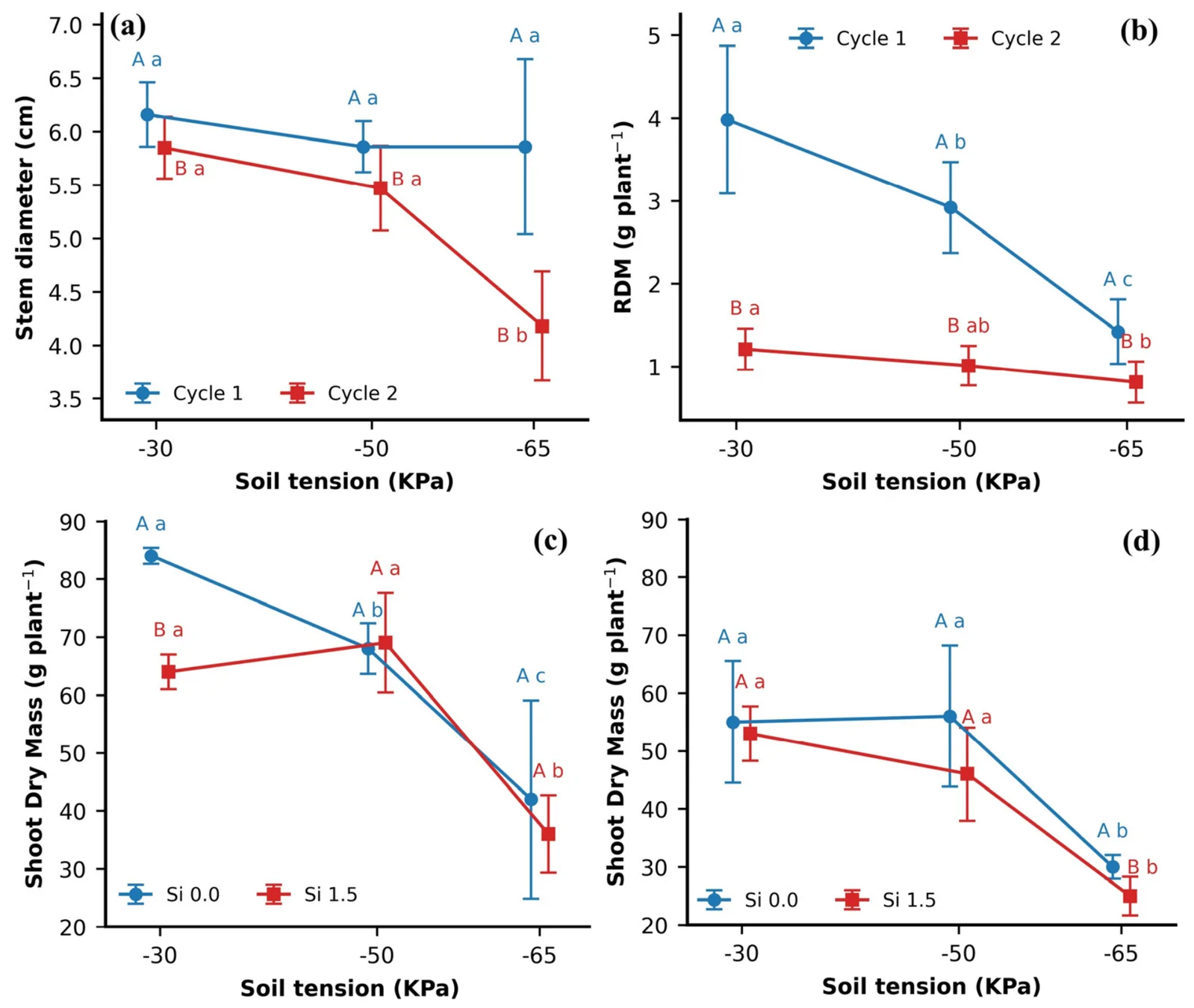
Stem diameter (**a**), root dry matter (RDM) (**b**), and shoot dry matter in cycle 1 (**c**) and cycle 2 (**d**) as a function of soil water tension for the significant interaction with cycle (**a**,**b**) and silicon fertilization ((**c**,**d**), g L^−1^). Lowercase letters compare means across soil water tensions, whereas uppercase letters compare silicon treatments within the same soil water tension. Means followed by the same letter do not differ significantly according to Tukey’s HSD test (*p* ≤ 0.05).

Stem diameter remained statistically unchanged across soil water tensions in cycle 1, ranging from 5.86 ± 0.54 to 6.16 ± 0.30 mm (Figure 1a). In cycle 2, however, stem diameter was lower than in cycle 1, with the lowest value observed at −65 kPa (4.18 ± 0.51 mm) (Figure 1a). Root dry matter decreased progressively as soil water tension increased from −30 to −65 kPa in both cycles, declining from 3.98 ± 0.89 to 1.42 ± 0.38 g plant^−1^ in cycle 1 and from 1.21 ± 0.25 to 0.81 ± 0.25 g plant^−1^ in cycle 2 (Figure 1b).

In cycle 1, shoot dry matter differed between silicon treatments only at −30 kPa, with greater biomass accumulation in plants not treated with silicon, reaching 84 ± 1.40 and 64 ± 3.01 g plant^−1^ for treatments without and with silicon, respectively (Figure 1c). In the absence of silicon, shoot dry matter decreased consistently as soil water tension increased. Conversely, silicon-treated plants showed similar shoot dry matter at −30 and −50 kPa. In cycle 2, silicon affected shoot dry matter only under severe water deficit conditions, at −65 kPa, where silicon-treated plants exhibited lower biomass accumulation than untreated plants, with values of 25 ± 3.32 and 30 ± 2.03 g plant^−1^, respectively (Figure 1d).

### 2.2. Physiological Measurements

Photosynthetic CO_2_ assimilation, stomatal conductance, transpiration rate, and relative water content showed significant interactions (*p* < 0.001) between crop cycle and soil water tension, whereas internal CO_2_ concentration (C_i_) was affected only by crop cycle (*p* = 0.014) (Table 3). Relative water content also showed a significant interaction between crop cycle and silicon application (*p* = 0.002).

**Figure 2:**
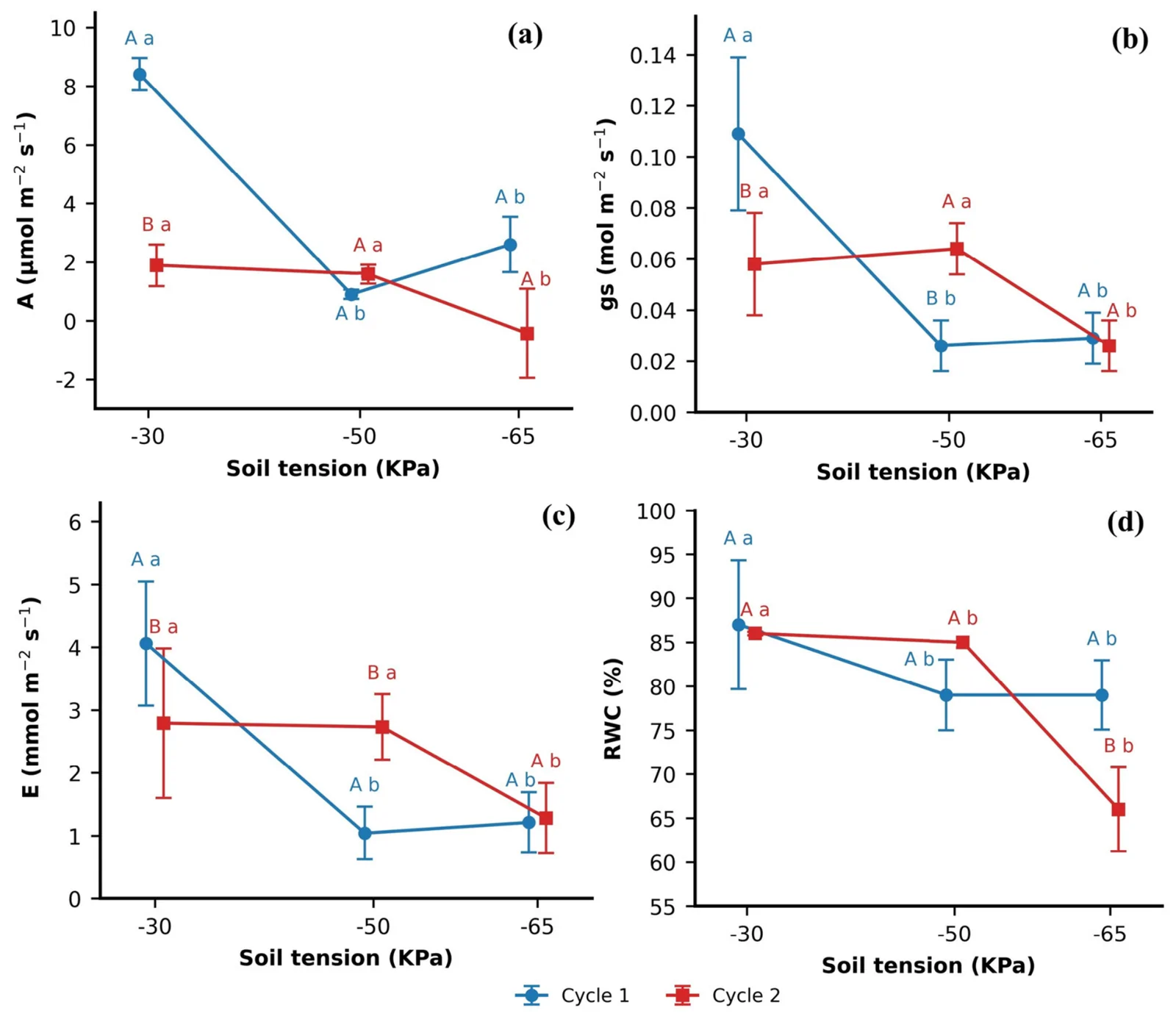
Photosynthetic CO_2_ assimilation (A) (**a**), stomatal conductance (g_s_) (**b**), transpiration (E) (**c**) and relative water content (RWC) (**d**) measured at 60 days after planting as a function of cycle and soil tension with significant interaction. Lowercase letters compare means across soil tension and uppercase letters compare lines in the same soil tension, where the same letters are not significantly different according to Tukey’s HSD test (*p* ≤ 0.05).

In cycle 1, photosynthetic CO_2_ assimilation decreased from 8.41 ± 1.70 μmol m^−2^ s^−1^ at −30 kPa to 0.90 ± 0.91 and 2.60 ± 1.73 μmol m^−2^ s^−1^ at −50 and −65 kPa, respectively (Figure 2a). In cycle 2, photosynthetic CO_2_ assimilation at −30 kPa was lower than that observed in cycle 1, reaching 1.89 ± 2.37 μmol m^−2^ s^−1^, whereas values at −50 and −65 kPa were similar between cycles. Within cycle 2, a significant reduction was observed only at −65 kPa, where photosynthetic CO_2_ assimilation reached −0.43 ± 2.67 μmol m^−2^ s^−1^, differing statistically from the values observed at −30 and −50 kPa (Figure 2a).

Stomatal conductance followed a similar pattern to photosynthetic CO_2_ assimilation. In cycle 1, stomatal conductance declined from 0.109 ± 0.03 to 0.026 ± 0.01 mol m^−2^ s^−1^ between −30 and −50 kPa, whereas in cycle 2 the reduction became more pronounced only at −65 kPa (Figure 2b). Transpiration showed the same trend as stomatal conductance, decreasing from 4.06 ± 1.00 and 2.79 ± 1.19 mmol m^−2^ s^−1^ at −30 kPa to 1.21 ± 0.48 and 1.28 ± 0.57 mmol m^−2^ s^−1^ at −65 kPa for cycles 1 and 2, respectively (Figure 2c). For relative water content, differences between cycles were observed only at −65 kPa, with cycle 1 maintaining higher values than cycle 2, 79 ± 3.87% versus 67 ± 8.72% (Figure 2d). Internal CO_2_ concentration was higher, on average, in cycle 2 than in cycle 1 (206 ± 129 versus 139 ± 49 ppm) (Table 4). Silicon application did not produce significant effects on photosynthetic CO_2_ assimilation, stomatal conductance, transpiration, or internal CO_2_ concentration (Table 3). Likewise, no significant differences were observed for relative water content according to Tukey’s test (Table 4) at 60 days after planting.

### 2.3. Plant Biochemistry

Total soluble carbohydrates showed a significant interaction among crop cycle, soil water tension, and silicon application (*p* = 0.036) (Table 5), whereas proline content was significantly affected by crop cycle and soil water tension. Considering only cycle 2, superoxide dismutase activity and malondialdehyde content were significantly affected by soil water tension, while pyrogallol peroxidase activity showed no significant response to either soil water tension or silicon fertilization (Table 5).

**Figure 3:**
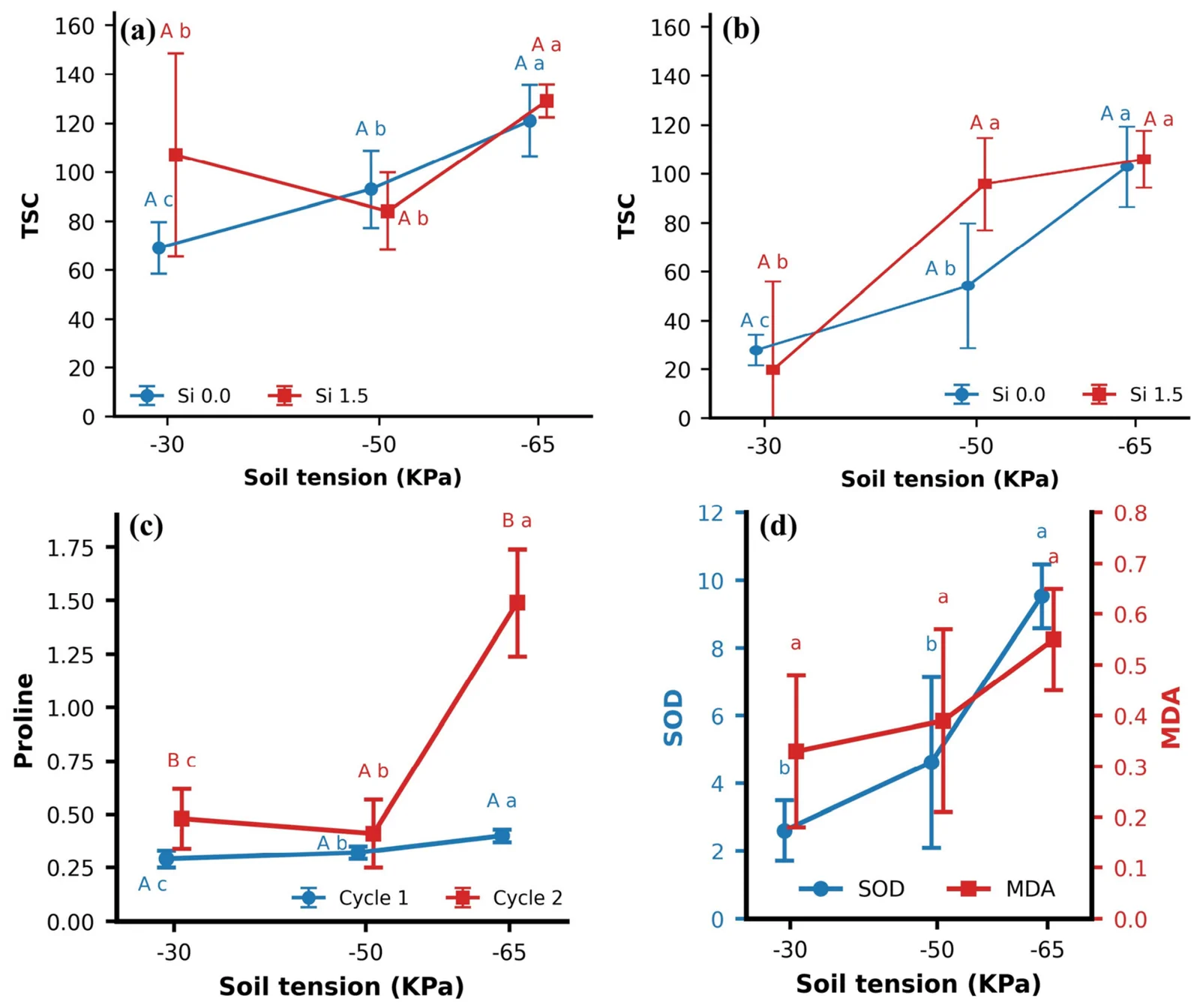
Total soluble carbohydrates (TSC) in cycle 1 (**a**) and cycle 2 (**b**), proline content (**c**), and superoxide dismutase activity (SOD) and malondialdehyde content (MDA) in cycle 2 (**d**), measured at 60 days after planting as affected by silicon application and soil water tension under significant interaction effects. Lowercase letters compare means across soil water tensions, whereas uppercase letters compare silicon treatments within the same soil water tension. Means followed by the same letter do not differ significantly according to Tukey’s HSD test (*p* ≤ 0.05).

In the absence of silicon fertilization, total soluble carbohydrates increased as soil water tension increased from −30 to −65 kPa, increasing from 69 ± 10.6 to 121 ± 14.5 mg g^−1^ DM in cycle 1 and from 27 ± 6.2 to 101 ± 16.2 mg g^−1^ DM in cycle 2 (Figure 3a,b). Overall, cycle 1 showed higher total soluble carbohydrate content than cycle 2, with mean values of 100 ± 28.1 mg g^−1^ DM and 69 ± 37.1 mg g^−1^ DM, respectively. However, silicon application promoted higher total soluble carbohydrate accumulation under water deficit conditions, especially at −65 kPa in cycle 1, reaching 129 ± 6.6 mg g^−1^ DM, and at −50 and −65 kPa in cycle 2, reaching values above 94 ± 18.6 mg g^−1^ DM (Figure 3a,b).

Proline content increased progressively with increasing soil water tension in both cycles. In cycle 1, proline increased from 0.29 ± 0.07 µmol g^−1^ DM at −30 kPa to 0.40 ± 0.20 µmol g^−1^ DM at −65 kPa, with significant differences among all soil tension levels. In cycle 2, proline followed a similar trend but reached substantially higher values under severe water deficit, attaining 1.49 ± 0.25 µmol g^−1^ DM at −65 kPa (Figure 3c).

Superoxide dismutase (SOD) activity and malondialdehyde (MDA) content increased under lower soil water availability (Figure 3d). SOD activity showed similar values at −30 and −50 kPa but increased significantly at −65 kPa, reaching 9.53 ± 0.95 U min^−1^ g^−1^ FM. MDA content increased numerically from 0.33 ± 0.16 nmol g^−1^ FM to 0.55 ± 0.11 nmol g^−1^ FM between −30 and −65 kPa, but this increase was not statistically significant.

Pyrogallol peroxidase (POD) activity did not differ among treatments or factors, with an overall mean of 0.0056 ± 0.0028 U mg^−1^ protein, although it showed a numerical increase from 0.004 ± 0.0013 U mg^−1^ protein at −30 kPa to 0.007 ± 0.0033 U mg^−1^ protein at −65 kPa.

### 2.4. Fruit Measurements

Fruit traits were evaluated only in cycle 2, in which most variables showed significant effects of soil water tension as an isolated factor, including average fruit weight, pulp thickness, fruit length, fruit diameter, pH, soluble solids, and fruit texture (Table 6). Silicon application significantly affected average fruit weight and fruit length. A significant interaction between soil water tension and silicon application was observed only for rind thickness, whereas the number of fruits per plant was not significantly affected by the evaluated factors (Table 6), with an overall mean of 2.21 ± 0.88 fruits per plant.

Average fruit weight showed a consistent response to soil water tension, differing significantly across all evaluated levels, with values of 0.398 ± 0.063, 0.211 ± 0.035, and 0.153 ± 0.051 kg at −30, −50, and −65 kPa, respectively (Table 7). Pulp thickness, fruit length, fruit diameter, and pH presented the highest values at −30 kPa, whereas −50 and −65 kPa were statistically similar (Table 7). For example, pulp thickness reached 41 ± 5.8 mm at −30 kPa and decreased to 32 ± 2.3 and 31 ± 3.7 mm at −50 and −65 kPa, respectively. Soluble solids (°Brix) and fruit texture showed significant reductions only under severe water deficit at −65 kPa, reaching values of 4.97 ± 0.43 °Brix and 9.8 ± 1.8 N, respectively, whereas at −30 kPa these variables reached 6.82 ± 0.47 °Brix and 12.1 ± 2.8 N (Table 7).

Average fruit weight and fruit length responded negatively to silicon application, with fruit weight decreasing from 0.281 ± 0.122 to 0.227 ± 0.112 kg and fruit length decreasing from 80 ± 13.4 to 75 ± 13.6 mm in treatments without and with silicon application, respectively (Table 7). Rind thickness showed a significant interaction between silicon application and soil water tension. Silicon application reduced rind thickness from 7.3 ± 2.36 mm in plants without silicon to 4.0 ± 0.64 mm in silicon-treated plants at −65 kPa (Table 7). Soil water tension did not significantly affect rind thickness in silicon-treated plants. In contrast, in the absence of silicon, the highest rind thickness values were observed at −30 and −65 kPa, which differed statistically from the intermediate tension level (−50 kPa) (Table 7).

## 3. Discussion

Cycle 1 was an important factor due to the absence of fruit production and the occurrence of disease with symptoms consistent with Zucchini lethal chlorosis virus (ZLCV) infection, resulting in plants failing to set fruit. In cycle 1, greater plant length was associated with increased vegetative biomass accumulation due to the absence of fruits, whereas cycle 2 presented an active reproductive sink. In this case, it reflected a shift in assimilate partitioning toward vegetative growth rather than fruit production, indicating alterations in the source–sink relationship caused by crop failure regarding fruit set [14]. The higher root dry mass in cycle 1 provides additional evidence for a substantial reconfiguration of assimilate partitioning. This is consistent with patterns reported in other cultivated cucurbits, when the plant reallocates photoassimilates toward vegetative organs, including expansion of the root system [15]. Furthermore, the 3 L pot volume may have imposed some degree of root restriction as plants developed, which can change plant water relations and root-to-shoot signaling [16].

The similar shoot dry matter observed at −30 and −50 kPa in silicon-treated plants during cycle 1 suggests a partial attenuation of moderate water restriction. However, the effects of silicon were not consistently beneficial across crop cycles or stress intensities. Overall, these responses indicate that increasing soil water tension progressively restricts vegetative growth and dry matter accumulation, particularly under severe water deficit conditions. This response is physiologically consistent with reduced leaf expansion, lower carbon assimilation, and impaired biomass partitioning under limited water availability [17].

Silicon application reduced plant length in both cycles by approximately 21% in cycle 1 and 14% in cycle 2. Ref. [18] reported in cucumber that potassium silicate fertilization also reduced productive parameters and leaf biomass and altered physiological and nutritional traits, including reduced leaf Ca content, in plants exposed to UV and drought. The phytotoxic effects observed for watermelon, a non-Si-accumulating species, induced growth abnormalities, morphological deformation, and severe phytotoxicity [19,20]. The foliar Si rate used in this study was 1.5 g L^−1^, representing 225 g ha^−1^. This concentration exceeds the standard range typically applied in foliar treatments for cucurbits, being between 0.125 and 0.5 g L^−1^ Si [19,20,21,22]. In melon, ref. [23] evaluated 0.3–1.2 g L^−1^ Si and reported favorable physiological and fruit-quality responses at intermediate concentrations. Si responses in Cucurbitaceae depend on dose, source, application method, and species, which may explain the inconsistent benefits, reflecting limited Si uptake and accumulation rather than true physiological non-responsiveness. The lower RWC observed in cycle 2 under −65 kPa indicates that plants were more affected than in cycle 1, associated with plant activity due to fruit growth, which was absent in cycle 1. The higher internal CO_2_ concentration combined with the lower photosynthetic CO_2_ assimilation observed in cycle 2 suggests that non-stomatal limitations likely accompanied stomatal restriction during the fruiting stage. These responses are consistent with previous studies on watermelon under water deficit conditions, reporting reductions in photosynthetic rate, stomatal conductance, and transpiration under the driest irrigation threshold, while fruit set and marketable yield were maximized when irrigation was initiated between −20 and −40 kPa [24,25].

Soil water tension influenced the water status of mini watermelon plants, as evidenced by the reduction in leaf RWC. This is a common response to water deficit in this crop [26] and reflects a reduction in available cellular water in leaves. The change in plant water status induced a water-saving strategy in mini watermelon during cycle 1, characterized by reductions in stomatal conductance, transpiration, and CO_2_ uptake [27]. This was demonstrated by simultaneous reductions in A, g_s_, and E with increasing soil water tension, indicating that stomatal closure was the main limitation to photosynthesis [28], as previously observed in watermelon under water shortage [26,29]. In cycle 2, however, the increased C_i_ and lower A suggest mesophyll limitation, probably due to damage to the photosynthetic apparatus [27].

In watermelon, ref. [30] observed that water deficit decreased proteins involved in photochemical reactions, diminishing linear electron transport activity. Notably, in cycle 2, net CO_2_ assimilation became negative (−0.43 µmol m^−2^ s^−1^) at −65 kPa. This represents a more severe physiological response than a simple reduction in photosynthetic rate, because net CO_2_ release exceeded photosynthetic CO_2_ fixation. Under mild to moderate water deficit, reductions in photosynthesis are generally dominated by stomatal and diffusional limitations; however, as drought severity increases, non-stomatal limitations involving photochemical activity, ATP and RuBP regeneration, and carbon metabolism become increasingly important. Accordingly, the negative A observed at −65 kPa suggests that photosynthetic impairment at this soil water tension was no longer attributable solely to restricted stomatal CO_2_ supply but also involved substantial metabolic limitation of carbon assimilation, even under a lower vapor pressure deficit in cycle 2 than in cycle 1. At this point, respiratory and photorespiratory CO_2_ release, combined with strongly suppressed carboxylation, resulted in a negative leaf carbon balance [31]. In this case, the severity of stress effects depends on the phenological stage of the plant, since water demand varies during the growth stages of watermelon [32]; in cycle 1, plants were still in the vegetative phase, whereas in cycle 2, plants were in the fruiting stage.

Previous studies emphasize that the effectiveness of silicon depends strongly on factors such as species, genotype, application method, and silicon formulation [33]. This dependence may explain the absence of significant Si effects on photosynthetic CO_2_ assimilation, stomatal conductance, transpiration, internal CO_2_ concentration, and relative water content in the present study.

Plant biochemistry showed that an increase in proline under reduced soil water tension agrees with the authors of [1], who reported that silicon application through nanoparticles improved proline accumulation in different plants. The accumulation of soluble carbohydrates and proline is a common response to water deficit [34,35]. Solute accumulation indicates a potential osmotic adjustment, a strategy that intensifies according to stress intensity. These compatible solutes contribute to maintaining cellular osmotic balance, ROS scavenging, and membrane stabilization, improving plant resilience to water deficit [36].

Proline is synthesized and stored in watermelon leaves during stress, with a portion being translocated toward the roots [37]. The increase in proline content, especially at severe soil tensions during cycle 2, suggests an amplified response to the combination of water stress and fruiting demand for carbon, as noted by [38]. Under severe stress, carbon resources are preferentially allocated to other protective mechanisms, leading to a decrease in soluble sugar concentrations [38,39]. In contrast, the present study indicated that under severe water deficit, soluble sugars reached their highest values, despite the decline in A during cycle 1 and the negative values observed in cycle 2.

The significant increase in SOD activity at −65 kPa indicates an upregulation of the primary enzymatic defense against stress [40]. In watermelon, activation of the enzymatic antioxidant system occurs in response to water deficit, especially through increased SOD activity [35], and the magnitude of this response may influence the extent of oxidative damage in plants [34]. SOD catalyzes the dismutation of superoxide radicals (O_2_•^−^) into H_2_O_2_, thereby limiting the accumulation of the highly reactive superoxide radical [41]. However, an effective antioxidant response requires coordinated detoxification of the H_2_O_2_ generated by SOD through downstream peroxide-scavenging systems. In the present study, POD activity did not increase significantly under severe water deficit, suggesting that enhanced superoxide dismutation was not accompanied by a proportional increase in the POD-mediated H_2_O_2_-scavenging response. This pattern suggests incomplete coordination of the measured enzymatic antioxidant response rather than a uniformly enhanced antioxidant defense.

POD represents only one component of H_2_O_2_ detoxification, which also involves catalase, ascorbate peroxidases, peroxiredoxins, and non-enzymatic redox systems [42]. Therefore, the present data do not allow direct inference of H_2_O_2_ accumulation. Nevertheless, increased SOD activity in the absence of POD upregulation could increase the demand placed on alternative H_2_O_2_-scavenging pathways. If H_2_O_2_ production exceeds cellular scavenging capacity, its accumulation, particularly in the presence of reduced transition metals, may favor the formation of highly reactive hydroxyl radicals and promote oxidation of membrane lipids. Under severe drought, progressive lipid peroxidation may increase membrane permeability and compromise the structural and functional integrity of cellular and chloroplast membranes, including components involved in photosynthetic electron transport [43].

Although MDA content increased numerically with increasing soil water tension, differences among treatments were not significant. Therefore, this numerical increase should not be interpreted as statistical evidence of enhanced lipid peroxidation or substantial membrane damage. Rather, it may be compatible with increased oxidative pressure that remained below the threshold required to produce a detectable increase in MDA at the time of sampling. Thus, membrane-protective and antioxidant mechanisms may still have been sufficient to limit measurable lipid peroxidation despite the stronger oxidative challenge imposed by severe water deficit.

Consequently, the combination of enhanced SOD activity, unchanged POD activity, and the numerical increase in MDA observed at −65 kPa is consistent with greater oxidative pressure on leaf tissues, although it does not demonstrate irreversible membrane injury [43]. This interpretation is also physiologically consistent with the decline in photosynthetic carbon assimilation observed under severe water deficit. Reduced CO_2_ availability under drought can increase the imbalance between light energy absorbed by the photosynthetic apparatus and its utilization in carbon fixation, favoring ROS generation in chloroplasts [44]. ROS homeostasis depends on the dynamic balance between their production and removal, and disruption of this balance during severe drought may shift ROS from their regulatory role toward oxidative effects [4]. Thus, the antioxidant response observed here may reflect increasing redox constraints accompanying the reduction in photosynthetic performance and, ultimately, dry-matter accumulation, rather than oxidative damage being considered the sole cause of these responses.

The responses observed for fruit traits show that fruit-related variables are modulated by genetic and agro-environmental factors [45]. In this case, soil water tension was the stronger factor than fruit weight and °Brix, where moderate (−50 kPa) and severe (−65 kPa) regimes yield commercially unviable fruit. The negative response of average fruit weight and fruit length to silicon application may depend on the application route, dose, and absorption dynamics of the studied species [46]. Further, foliar Si significantly reduced fruit rind thickness from 7.3 mm to 4.0 mm under −65 kPa water tension, which increases edible pulp proportion, increasing fruit fragility during shipping and handling.

## 4. Materials and Methods

The research was conducted in two cycles, cultivating the watermelon cv. Sugar Baby (*Citrullus lanatus*) in a greenhouse located at (16°59′61″23 S and 49°27′99″06 W). The plantings were on 24 August 2022 for the first cycle and on 3 February 2023 for the second cycle. During cycles 1 and 2, weather station in the greenhouse recorded air temperature ranged from 20.5–32.2 °C and 21.0–30.2 °C, relative humidity from 25.8–95.3% and 39.4–100%, mean solar radiation was 15.20 and 13.80 MJ m^−2^ day^−1^, and mean (maximum) vapor pressure deficit was 1.36 (3.57) and 1.00 (2.60) kPa, respectively. The experimental unit consisted of cultivation in 3-L pots, filled with soil samples with the following attributes: pH = 5.3; P (Mehl) = 1.5 mg dm^−3^; H + Al = 1.9 cmolc dm^−3^; K = 77 mg dm^−3^; Ca = 280 mg dm^−3^; Mg = 60.75 mg dm^−3^; organic matter = 1.2; cation exchange capacity (CEC) = 4.0 cmolc dm^−3^; sand = 43%, silt = 15%, and clay = 42%. After homogenizing the soil, sowing took place directly in the pots, aiming for one plant per pot.

The experiment was designed in randomized blocks, including four replications, with treatments arranged in a 2 × 3 × 2 factorial scheme, corresponding to two crop growing cycles, three water tensions in the soil: −30 kPa, corresponding to well-watered conditions without water deficit, −50 kPa, representing moderate water deficit, and −65 kPa, representing severe water deficit; and the presence or absence of Si. Si was applied to the leaves using potassium silicate (137.0 g L^−1^ Si and 164.4 g L^−1^ K_2_O) stabilized with 70% sorbitol, with a final pH around 11. The concentrations were 0.0 (control) and 1.5 g L^−1^, considering an application volume of 150 L ha^−1^, with weekly applications from the initial growth stage (30 days after emergence) until fruiting, corresponding to a total Si dose of 225 g ha^−1^. A 50 mL solution per plant containing K supplied as KCl was applied to the Si-free treatments to equalize potassium availability and eliminate potential confounding effects of this nutrient.

Soil water potential was monitored using SoilWatch 10 capacitance sensors (PINO-TECH, Stargard, Poland) installed at a depth of 10 cm below the soil surface and connected to Bristom DS−210 data loggers (Briston, Goiânia, Brazil). One sensor was used per pot, which was calibrated prior to installation according to the methodology described by [47]. Irrigation was carried out by drip, starting when the soil water potential reached −30, −50, and −65 kPa, ending, respectively, when the potential reached −15, −25, and −35 kPa, controlled automatically by the data logger system. Water deficit began when the plants reached the 3rd definitive leaf.

Biometric analyses were carried out at the end of the cycle, including plant length, stem diameter, shoot dry mass, and root dry mass. Plant length was measured from the base of the plant to the apex of the main stem using a tape measure, and the diameter of the stem was measured at the base of the plant using a manual caliper [48]. Shoot and root dry mass were measured by weighing biomass after oven-drying at 65 °C until constant mass [49].

In the first cycle, symptoms consistent with Zucchini lethal chlorosis virus (ZLCV) infection were observed, including foliar chlorosis and deformation, and affected plants failed to set fruit. In the second cycle, the post-harvest parameters were evaluated, including fruits per plant (NFP), average fruit weight (AFW), rind thickness (RT), pulp thickness (PT), fruit length (FL), fruit diameter (FD), hydrogen potential (pH), soluble solids (°Brix), and texture. ST comprises the region from the epidermis to the transition zone of pulp color (white-red), measured with a manual caliper, and PT is from the transition zone (white-red) to the center of the fruit [50]. FL and FD were measured with a manual caliper, including measurements from the peduncle to the apex of the fruit and the middle part of the fruit [50]. pH and °Brix were measured from watermelon juice.

Photosynthetic CO_2_ assimilation (A), stomatal conductance (g_s_), transpiration (E), and internal CO_2_ concentration (C_i_) were measured during the fruiting stage at both cycles, occurring around 60 days after emergence. Assessments were conducted between 7:00 and 9:00 a.m. on the fourth fully expanded and healthy leaf from the base to the apex, using an infrared gas analyzer (IRGA, LI-6400XT, Li-Cor Biosciences^®^, Lincoln, NE, USA). During measurements, photosynthetic photon flux density (PPFD) was maintained at 1000 µmol m^−2^ s^−1^, block temperature at 25 °C, reference CO_2_ concentration at 400 µmol mol^−1^, and airflow rate at 500 µmol s^−1^. The relative water content (RWC) was measured according to [51] on ten fully expanded leaf disks (1.15 cm^2^).

Total soluble carbohydrates (TSC) were assayed in fully expanded leaves from the main branch collected between 7:00 and 9:00 am. The leaves were heated in a microwave oven for 1 min for enzymatic inactivation and were oven-dried at 60 °C for 24 h. The dried leaves were ground in a mortar and pestle to a fine powder. Soluble carbohydrates were meticulously extracted from 30 mg of the powder with 1 mL of aqueous ethanol (80%). After homogenization, the mixtures were set in a water bath at 80 °C for 15 min, then centrifuged at 10,000 rpm for 10 min for supernatant collection. The process was repeated four times. For the last extraction, the process was repeated but with distilled water. Finally, all supernatants were collected and pooled [52]. TSC were quantified by the phenol-sulfuric acid method, using glucose as a standard [53]. The absorbance was determined at 490 nm in a spectrophotometer (BEL 1105) (BEL Engineering, Monza, Italy) and measured in triplicate. Proline content was quantified following [54], with absorbance read at 520 nm in triplicate using a spectrophotometer. Proline concentration was calculated on the dry mass basis (μmol g^−1^ MS). Additionally, in the second cycle, malondialdehyde content (MDA), pyrogallol peroxidase activity (POD), and superoxide dismutase activity (SOD) were evaluated [55].

The data were subjected to analysis of variance (ANOVA), and when significant, means were compared using Tukey’s test at a 5% probability level (*p* ≤ 0.05), using AgroEstat^®^ statistical software (Version 1.1). The assumptions of normality and homogeneity of variances were verified prior to analysis.

## 5. Conclusions

Soil water tension was the main determinant of mini-watermelon performance across the two production cycles, whereas the tested foliar silicon treatment produced modest and inconsistent responses. Maintaining soil water tension near −30 kPa favored biomass accumulation, leaf gas exchange, and fruit growth and quality. Increasing tension to −50 kPa substantially reduced fruit mass, although some quality attributes, particularly soluble solids, were relatively preserved. At −65 kPa, plants exhibited severe physiological impairment, including marked reductions in carbon assimilation, increased accumulation of proline and soluble carbohydrates, and pronounced losses in fruit size, sweetness, and texture. In cycle 2, the negative net CO_2_ assimilation observed at −65 kPa further indicated that severe water deficit imposed substantial non-stomatal limitations and prevented the maintenance of a positive leaf carbon balance. Foliar Si affected specific traits, including plant length, carbohydrate accumulation, and rind thickness, but did not consistently improve gas exchange, relative water content, fruit number, or the main commercial fruit-quality attributes. Therefore, under the conditions evaluated, the applied Si dose and application program were insufficient to provide consistent mitigation of drought effects. Further studies should evaluate a broader range of Si doses and application strategies, larger rooting volumes, and multiple production cycles with complete fruit development to explore the potential of foliar Si as a drought-mitigation strategy in mini-watermelon.

## Figures and Tables

**Table 1:** *p*-value from analyses of variance applied for plant length (PL), stem diameter (ST), shoot dry matter (SDM), and root dry matter (RDM) at maturity as a function of cycle, soil tension, and silicon and their interaction.

Factors	PL		SD		SDM		RDM	
	*p*-Value							
Cycle	<0.001	***	<0.001	***	<0.001	***	<0.001	***
Soil tension	<0.001	***	<0.001	***	<0.001	***	<0.001	***
Silicon	<0.001	***	0.002	**	0.003	**	0.056	ns
Block	0.410	ns	0.052	ns	0.017	*	0.276	ns
Cycle × Soil tension	0.097	ns	<0.001	***	0.328	ns	<0.001	***
Cycle × Silicon	0.019	*	0.471	ns	0.544	ns	0.016	*
Soil tension × Silicon	0.376	ns	0.051	ns	0.409	ns	0.933	ns
Cycle × Soil tension × Silicon	0.705	ns	0.769	ns	0.031	*	0.448	ns

ns = not significant; * = *p* < 0.05; ** = *p* < 0.01; *** = *p* < 0.001.

**Table 2:** Mean followed by standard deviation. Tukey test applied to plant length and root dry matter (RDM) for the interaction of cycle and silicon and stem diameter for silicon fertilization.

Cycle	Silicon (g L^−1^)	
	0	1.5
Plant length (cm)		
1	235 ± 51 Aa	186 ± 51 Ab
2	132 ± 48 Ba	114 ± 49 Bb
RDM (g plant^−1^)		
1	3.07 ± 1.31 Aa	2.48 ± 1.14 Aa
2	0.97 ± 0.27 Ba	1.05 ± 0.32 Ba
Stem diameter (mm)		
Both	5.75 ± 0.77 a	5.38 ± 0.79 a

Lowercase compares means in the row and uppercase compares means in the column. Means sharing the same letters are not significantly different according to Tukey’s HSD test (*p* ≤ 0.05).

**Table 3:** *p*-values from the analysis of variance for photosynthetic CO_2_ assimilation (A), stomatal conductance (g_s_), transpiration rate (E), internal CO_2_ concentration (C_i_), and relative water content (RWC) at 60 days after planting, as affected by crop cycle, soil water tension, silicon application, and their interactions.

Factors	A		g_s_		E		C_i_		RWC	
	*p*-Value									
Cycle	<0.001	***	0.286	ns	0.477	ns	0.014	*	0.085	ns
Soil tension	<0.001	***	<0.001	***	<0.001	***	0.352	ns	<0.001	***
Silicon	0.625	ns	0.189	ns	0.292	ns	0.349	ns	0.092	ns
Block	0.394	ns	0.510	ns	0.576	ns	0.139	ns	0.163	ns
Cycle × Soil tension	<0.001	***	<0.001	***	<0.001	***	0.299	ns	<0.001	***
Cycle × Silicon	0.371	ns	0.250	ns	0.329	ns	0.098	ns	0.002	**
Soil tension × Silicon	0.638	ns	0.928	ns	0.865	ns	0.127	ns	0.281	ns
Cycle × Soil tension × Silicon	0.305	ns	0.744	ns	0.713	ns	0.162	ns	0.586	ns

ns = not significant; * = *p* < 0.05; ** = *p* < 0.01; *** = *p* < 0.001.

**Table 4:** Mean followed by standard deviation and Tukey test applied to relative water content (RWC) for the interaction of cycle and silicon, and internal CO_2_ concentration (C_i_) for cycle.

Cycle	Silicon	
	0	1.5
RWC (%)		
1	82 ± 4.63 Aa	83 ± 4.85 Aa
2	79 ± 9.15 Aa	79 ± 8.15 Aa
C_i_ (ppm)		
1	139 ± 49 B	
2	206 ± 129 A	

Lowercase letters compare means in the row and uppercase letters compare means in the column, where the same letters are not significantly different according to Tukey’s HSD test (*p* ≤ 0.05).

**Table 5:** *p*-values from the analysis of variance for total soluble carbohydrates (TSC), proline, superoxide dismutase activity (SOD), pyrogallol peroxidase activity (POD), and malondialdehyde content (MDA) at 60 days after planting, as affected by crop cycle, soil water tension, silicon fertilization, and their interactions.

Factor	TSC		Proline		SOD		POD		MDA	
	*p*-Value									
Cycle	<0.001	***	<0.001	***						
Soil tension	<0.001	***	<0.001	***	<0.001	***	0.090	ns	0.020	*
Silicon	0.017	*	0.443	ns	0.100	ns	0.440	ns	0.750	ns
Block	0.297	ns	0.845	ns	0.470	ns	0.080	ns	0.070	ns
Cycle × Soil tension	0.019	*	<0.001	***						
Cycle × Silicon	0.686	ns	0.789	ns						
Soil tension × Silicon	0.463	ns	0.771	ns	0.650	ns	0.970	ns	0.490	ns
Cycle × Soil tension × Silicon	0.036	*	0.592	ns						

ns = not significant; * = *p* < 0.05; *** = *p* < 0.001. SOD, POD, and MDA were evaluated only in the second cycle.

**Table 6:** *p*-values from the analysis of variance for number of fruits per plant (NFP), average fruit weight (AFW), rind thickness (RT), pulp thickness (PT), fruit length (FL), fruit diameter (FD), hydrogen potential (pH), soluble solids (°Brix), and fruit texture at maturity, as affected by soil water tension, silicon application, and their interaction.

Factor	NFP		AFW		RT		PT		FL	
	*p*-Value									
Block	0.325	ns	0.073	ns	0.210	ns	0.377	ns	0.139	ns
Soil tension	0.955	ns	<0.00	***	0.035	*	0.005	**	<0.001	***
Silicon	0.532	ns	0.005	**	0.026	*	0.485	ns	0.039	*
Silicon × Tension	0.872	ns	0.971	ns	0.027	*	0.853	ns	0.689	ns
Factor	FD		pH		Brix		Texture			
	*p*-value									
Block	0.223	ns	0.546	ns	0.974	ns	0.170	ns		
Soil tension	<0.001	***	0.008	**	<0.001	***	0.002	**		
Silicon	0.423	ns	0.195	ns	0.572	ns	0.450	ns		
Silicon × Tension	0.991	ns	0.614	ns	0.076	ns	0.826	ns		

ns = not significant; * = *p* < 0.05; ** = *p* < 0.01; *** = *p* < 0.001.

**Table 7:** Mean followed by standard deviation and Tukey test applied to average fruit weight (AFW), rind thickness (RT), pulp thickness (PT), fruit length (FL), fruit diameter (FD), hydrogen potential (pH), soluble solids (°Brix), and texture at maturity in function soil tension and silicon and their interaction.

Soil Tension	AFW		PT		FL		FD		pH	°Brix		Texture
	kg		mm									N
−30	0.398 ± 0.063 a		41 ± 5.8 a		94 ± 5.4 a		97 ± 2.9 a		6.88 ± 0.24 a	6.82 ± 0.73 a		12.1 ± 2.8 a
−50	0.211 ± 0.035 b		32 ± 2.3 b		70 ± 9.3 b		76 ± 13.2 b		6.43 ± 0.30 b	6.20 ± 0.47 a		10.9 ± 1.8 ab
−65	0.153 ± 0.051 c		31 ± 3.7 b		69 ± 5.0 b		78 ± 4.1 b		6.28 ± 0.24 b	4.97 ± 0.43 b		9.8 ± 1.8 b
Silicon		Soil tension						AFW			FL	
		−30		−50		−65		kg			mm	
		RT										
		mm										
0		6.5 ± 0.33 Aab		4.3 ± 0.44 Ab		7.3 ± 2.36 Aa		0.281 ± 0.122 a			80 ± 13.4 a	
1.5		5.9 ± 0.34 Aa		4.9 ± 0.66 Aa		4.0 ± 0.64 Ba		0.227 ± 0.112 b			75 ± 13.6 b	

Lowercase letters compare means in the row and uppercase letters compare means in the column, where the same letters are not significantly different according to Tukey’s HSD test (*p* ≤ 0.05).

## Data Availability

The data presented in this study are available upon request from the first author. The data are not publicly available due to the data are securely deposited in the Central Library Repository of Federal University of Goiás but are not publicly available at this time because the comprehensive results are currently undergoing the publication process.
